# Antimicrobial Susceptibility Testing of Porcine Bacterial Pathogens: Investigating the Prospect of Testing a Representative Drug for Each Antimicrobial Family

**DOI:** 10.3390/antibiotics11050638

**Published:** 2022-05-10

**Authors:** Anna Vilaró, Elena Novell, Vicens Enrique-Tarancon, Jordi Balielles, Lourdes Migura-García, Lorenzo Fraile

**Affiliations:** 1 Grup de Sanejament Porcí, 25192 Lleida, Spain; micro@gsplleida.com (A.V.); elena@gsplleida.net (E.N.); vicens@gsplleida.net (V.E.-T.); jordi@gsplleida.net (J.B.); 2 Unitat Mixta d’Investigació IRTA-UAB en Sanitat Animal, Centre de Recerca en Sanitat Animal (CReSA), 08193 Bellaterra, Spain; lourdes.migura@irta.cat; 3 IRTA, Programa de Sanitat Animal, Centre de Recerca en Sanitat Animal (CReSA), 08193 Bellaterra, Spain; 4 Departament de Ciència Animal, ETSEA, University of Lleida-Agrotecnio, 25198 Lleida, Spain

**Keywords:** antimicrobial, susceptibility testing, MIC, porcine bacterial pathogen

## Abstract

Antimicrobial susceptibility testing is necessary to carry out antimicrobial stewardship but a limited number of drugs belonging to each antimicrobial family has to be tested for technical limitations and economic resources. In this study, we have determined the minimal inhibitory concentration, using microdilution following international standards (CLSI), for 490 *Actinobacillus pleuropneumoniae*, 285 *Pasteurella multocida*, 73 *Bordetella bronchiseptica*, 398 *Streptococcus suis* and 1571 *Escherichia coli* strains from clinical cases collected in Spain between 2018 and 2020. The antimicrobial susceptibility pattern was deciphered using a principal component analysis for each bacterium and a matrix correlation (high > 0.8, medium 0.5–0.8 and low < 0.5) was obtained for each pair of antimicrobials. No significant associations were observed between MIC patterns for different antimicrobial families, suggesting that co-selection mechanisms are not generally present in these porcine pathogens. However, a high correlation was observed between the fluroquinolones (marbofloxacin and enrofloxacin) for all mentioned pathogens and for ceftiofur and cefquinome for *E. coli* and *S. suis*. Moreover, a significant association was also observed for tetracyclines (doxycycline and oxytetracycline) and *B. bronchiseptica* and tildipirosin/tulathromycin for *P. multocida*. These results suggest that generally, a representative drug per antimicrobial class cannot be selected, however, for some drug–bug combinations, MIC values from one representative drug could be extrapolated to the whole antimicrobial family.

## 1. Introduction

The Porcine Respiratory Disease Complex (PRDC), systemic disorders due to *Streptococcus suis* (*S. suis*) infections and post-weaning diarrhea (PWD) are some of the most challenging diseases affecting the pig industry worldwide [1,2,3]. PRDC is a syndrome that results from a combination of infectious (bacteria and viruses) and non-infectious factors. *Actinobacillus pleuropneumoniae* (APP), *Pasteurella multocida (P. multocida)*, *Mycoplasma hyopneumoniae*, *Bordetella bronchiseptica* (*B. bronchiseptica*) and *Glaesserella* (*Haemophilus*) *parasuis* are the most common bacterial agents involved [1,4,5]. As a general approach, swine preventive medicine programs should be based on applying measures to control PRDC in a cost-effective way, such as improving environmental conditions, decrease density and stressors, combined with vaccination against the major viral and bacterial infectious etiologic factors adapted in a case-by-case situation [1,5]. However, if such measures are not in place or fail, the use of antimicrobials may be necessary [4].

In the case of *S. suis*, disease outbreaks mainly occur after weaning when maternal antibodies wane. The mortality rate can be as high as 30%, but less severe manifestations of the disease include polyarthritis, meningitis, endocarditis or pneumonia during the nursery period [3,6,7]. Up to date, there is not registered vaccine for *S. suis*. Thus, prevention is mainly based on biosecurity, hygiene measures with all-in all-out herd management reducing the spread of the pathogen, and the use of autogenous vaccines. However, when outbreaks occur, antimicrobials are commonly prescribed, increasing the risk of emergence of antimicrobial resistance (AMR) [8,9].

*Escherichia coli* is the main causative agent of PWD, affecting piglets after weaning. It is an economically important enteric disease causing significant financial losses to the pig sector. PWD is characterized by a profuse diarrhea, dehydration, significant mortality and loss of body weight in surviving pigs [10,11]. When clinical signs appear, prescription of antimicrobials is the only solution to control the spread of the disease within the herd [6,7,12]. 

The use of antimicrobials with a therapeutic or metaphylactic purpose may be necessary to control the relevant pathogens involved in PRDC, *S. suis* infections and PWD [6,7,13,14]. In particular, the objective of antimicrobial therapy is to provide an effective drug to obtain a fast clinical recovery from the infection, reducing the probability of generating AMR bacteria [15]. Thus, it is essential for the veterinarian to confirm by laboratory analysis the etiology of the disease, and determine the antimicrobial susceptibility of the pathogen. In both veterinary and human medicine, antimicrobial susceptibility testing (AST) data can help to predict the clinical outcome of antimicrobial treatment [16], allowing a rational choice of the drug to treat a particular bacterial infection [17,18]. Antimicrobial susceptibility is usually measured by the minimum inhibitory concentration (MIC), which is the lowest concentration that stops in vitro growth of the targeted bacteria. Still, suitable clinical breakpoints (CBPs) must be available for each pair of bacterial pathogen/antimicrobial [19] to interpret the MIC data once available, and to foresee the clinical outcome after treatment. Unfortunately, there are limited infection-specific and host-specific CBPs for porcine pathogens, especially for infections due to *S. suis* and *E. coli* among others. This critical point makes extremely challenging to compare available data on antimicrobial resistance for porcine pathogens [6], and makes urgent the need to set up a common method to determine the antimicrobial susceptibility pattern of pathogens of veterinary interests in Europe, as recently published [20].

When optimizing the panel of antimicrobials to be tested for each targeted bacteria, one of the critical points is to establish a balanced number of antimicrobial drugs representing all of the different antimicrobial families, without hampering the laboratory routine and exceeding economic cost [19]. For some combination of bacterial species and antimicrobials, such as polymyxins and *E. coli*, clearly, colistin is the best representative drug of the family to test [21], but when it comes to fluoroquinolones, beta-lactams, aminoglycosides and macrolides, there are several possible drugs to select within each family, but testing all of them can exceed the load of work in routine diagnostics and make the cost of analysis unaffordable [19,20]. Hence, the aim of this study was to investigate MIC data retrieved from our diagnostic laboratory for bacterial pathogens with relevance in pig production involved in PRDC (APP, *P. multocida* and *B. bronchiseptica*), *S. suis* and PWD (*E. coli*), to determine concurrent MIC patterns between different antimicrobial families and within the same family to optimize the number of antimicrobials to be tested in a diagnostic laboratory.

## 2. Results

### 2.1. Clinical Samples

From 2018 to 2020, 1060 samples were received from isowean, wean-to-finish and fattening farms suffering from clinical respiratory disease associated with the PRDC. Additionally, 496 and 1980 samples were received from sow, isowean and wean-to-finishing farms suffering clinical sings compatible with *S. suis* (SS) infection or PWD, respectively. In the case of sow farms, the samples were obtained from their nursery facility. Bacterial isolation for respiratory pathogens (APP, *P. multocida* (PM) and *B. bronchiseptica* (BB)) was possible in 80% (848/1060) of the cases, furthermore in 20%, it was possible to isolate more than one bacterial species from the same sample. Bacterial isolation of *S. suis* and *E. coli* (EC) was possible in 79.6% (398/496) and 79.3% (1571/1980) of the samples associated to systemic and digestive disorders, respectively. Finally, in 5% of the digestive samples, it was possible to isolate more than one bacterial species, generally *Salmonella* spp.

### 2.2. MIC Range, MIC50 and MIC90 for the Porcine Pathogens

MIC range, MIC_50_, MIC_90_ for 490, 285, 73, 398 and 1571 strains of APP, PM, BB, SS and EC are described in Table 1, Table 2 and Table 3, respectively. In general, a wide range of MIC was observed for each drug–bug combination with the exception of beta-lactams (amoxicillin and ceftiofur) and tiamulin for BB. Moreover, the MIC distributions were very different not only between drugs but also within each antimicrobial family for all the studied pathogens, with the exception of quinolones (enrofloxacin and marbofloxacin) for all the studied pathogens, tildipirosin and tulathromycin for PM, doxycycline and oxytetracycline for BB, amoxicillin and ampicillin for SS and ceftiofur and cefquinome for SS and EC.

### 2.3. Multivariate Analysis of MIC for Twelve Antimicrobials in Each Porcine Pathogen

#### 2.3.1. *Actinobacillus pleuropneumoniae* (APP)

**Figure 1:**
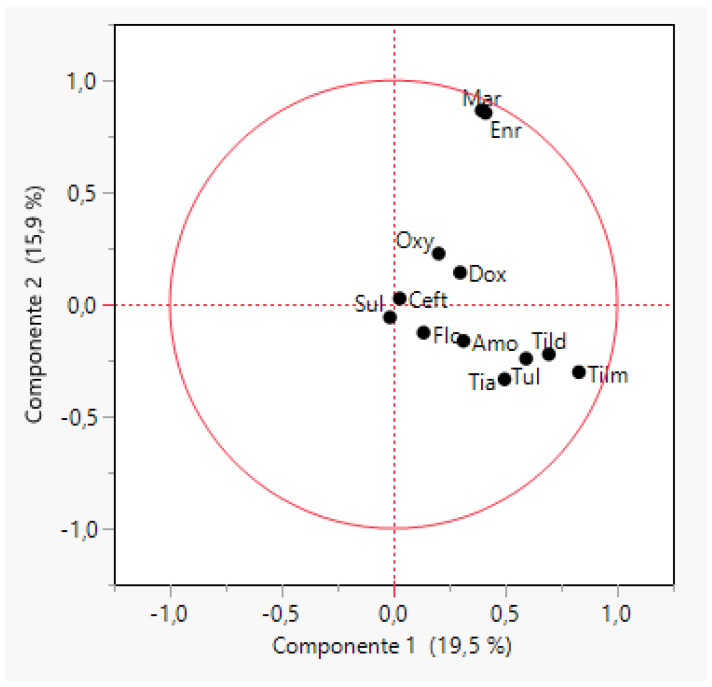
Biplot obtained through a principal component analysis of MIC values for twelve antimicrobials and 490 strains of *Actinobacillus pleuropneumoniae* (APP). The antimicrobials tested were amoxicillin (Amo), ceftiofur (Ceft), doxycycline (Dox), enrofloxacin (Enr), florfenicol (flo), marbofloxacin (Mar), oxytetracycline (Oxy), sulfamethoxazole/trimethoprim (Sul), tiamulin (Tia), tildipirosin (Tild), tilmicosin (Tilm) and tulathromycin (Tul).

The first two principal components could explain 35.4% of the variance for the MIC values of APP against all the tested antimicrobials. Sulphonamides and cephalosporins had characteristics corresponding to the most frequent MIC values (X = 0, Y = 0 in the graph) whereas quinolones (enrofloxacin and marbofloxacin) and macrolides (tilmicosin, tildipirosin and tulathromycin) showed the less frequent values, far from the origin of the axes (Figure 1). In general, a low correlation between each pair of antimicrobials was observed with the exception of enrofloxacin and marbofloxacin MIC values (R = 0.92), and intermediate correlation (R = 0.64) for the combination tilmicosin–tildipirosin, and doxycycline–oxytetracycline (R = 0.58). APP isolates were grouped into 19 clusters (Figure S1 in Supplementary Materials) according to their MIC values but most of the isolates (61.8%) were grouped in six clusters and seven clusters included only 8.8% of the remaining isolates.

#### 2.3.2. *Pasteurella multocida*

**Figure 2:**
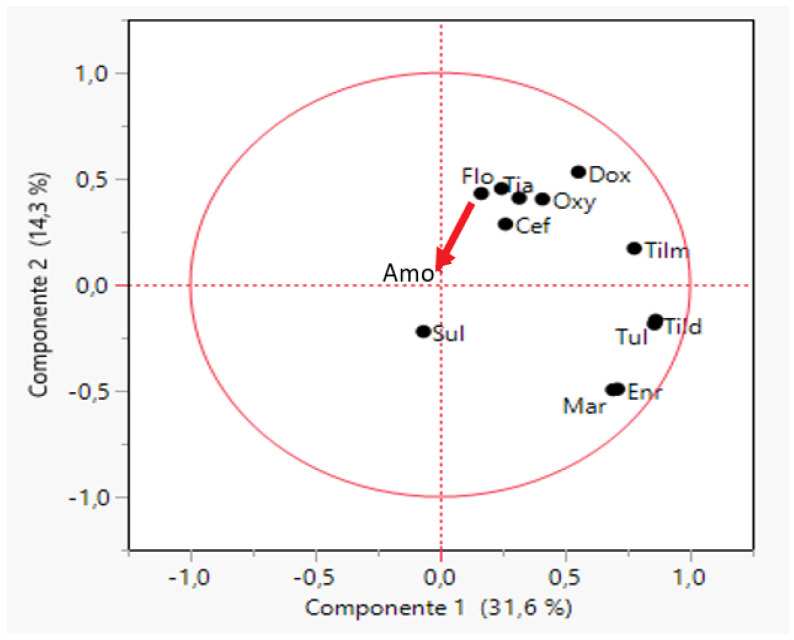
Biplot obtained through a principal component analysis of MIC values for twelve antimicrobials and 285 strains of *Pasteurella multocida* (PM). The antimicrobials tested were amoxicillin (Amo), ceftiofur (Ceft), doxycycline (Dox), enrofloxacin (Enr), florfenicol (flo), marbofloxacin (Mar), oxytetracycline (Oxy), sulfamethoxazole/trimethoprim (Sul), tiamulin (Tia), tildipirosin (Tild), tilmicosin (Tilm) and tulathromycin (Tul).

The first two principal components could explain 45.9% of the variance for the MIC values of *P. multocida* against all the tested antimicrobials. All the antimicrobials tested showed very variable MIC values, locating all of them far from the origin of the axes in the plot (Figure 2). In general, low correlation between each pair of antimicrobials was observed with the exception of a high association between enrofloxacin and marbofloxacin MIC values (R = 0.96) and tildipirosin and tulathromycin (R = 0.98). Tilmicosin–tildipirosin (R = 0.67), tilmicosin–tulathromycin (R = 0.68) and doxycycline–oxytetracycline (R = 0.58) exhibited intermediate correlation. Isolates of *P. multocida* were grouped into 15 clusters (Figure S2 in Supplementary Materials) according to their MIC values, but most of them (80.1%) were grouped in four clusters, whereas seven clusters included only 5% of the isolates.

#### 2.3.3. *Bordetella bronchiseptica*

**Figure 3:**
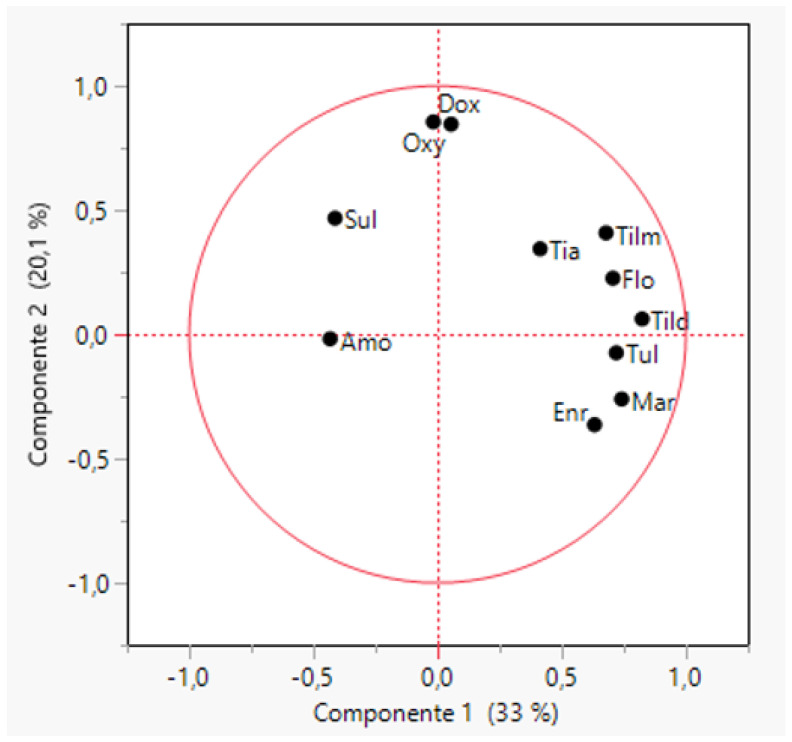
Biplot obtained through a principal component analysis of MIC values for twelve antimicrobials and 73 strains of *Bordetella bronchiseptica* (BB). The antimicrobials tested were amoxicillin (Amo), ceftiofur (Ceft), doxycycline (Dox), enrofloxacin (Enr), florfenicol (flo), marbofloxacin (Mar), oxytetracycline (Oxy), sulfamethoxazole/trimethoprim (Sul), tiamulin (Tia), tildipirosin (Tild), tilmicosin (Tilm) and tulathromycin (Tul).

The first two principal components could explain 53.4% of the variance for the MIC values against all the tested antimicrobials (Figure 3). Again, a low correlation between each pair of antimicrobials was observed with the exception of enrofloxacin and marbofloxacin MIC values (R = 0.94) and doxycycline–oxytetracycline (R = 0.84). Intermediate correlations (R = 0.5–0.7) were obtained for each pair of macrolides tested in the panel (tildipirosin, tilmicosin and tulathromycin). *B. bronchiseptica* isolates were grouped into eight clusters (Figure S3 in Supplementary Materials) according to their MIC values but most of the strains (73.7%) were grouped in three clusters, and three extra clusters included only 8.1% of the strains.

#### 2.3.4. *Streptococcus suis*

**Figure 4:**
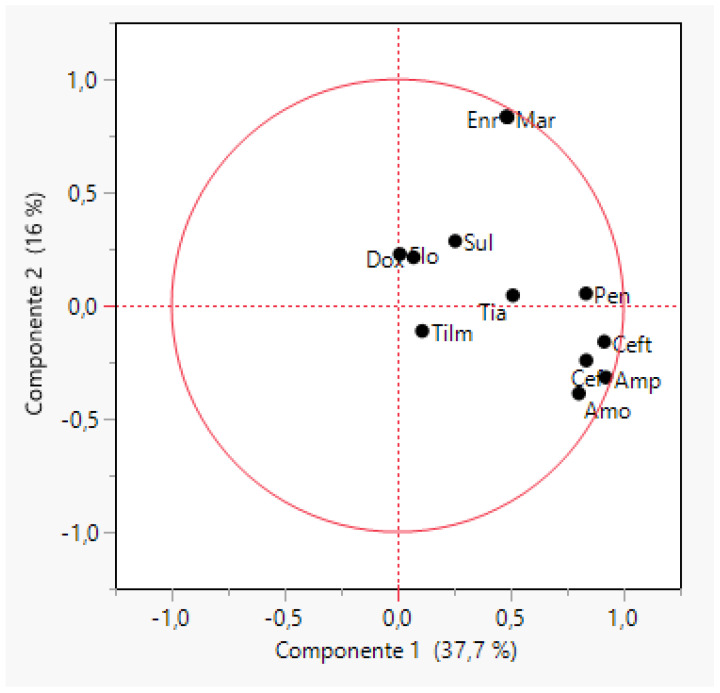
Biplot obtained through a principal component analysis of MIC values for twelve antimicrobials and 398 strains of *Streptococcus suis*. The antimicrobials tested were ampicillin (Amp), amoxicillin (Amo), cefquinome (Cef), ceftiofur (Ceft), doxycycline (Dox), enrofloxacin (Enr), florfenicol (flo), marbofloxacin (Mar), penicillin G, sulfamethoxazole/trimethoprim (Sul), tiamulin (Tia), and, tilmicosin (Tilm).

The first two principal components could explain 53.7% of the variance for the MIC values of *S. suis* against all the tested antimicrobials. Tilmicosin and doxycycline had characteristics corresponding to the most frequent MIC values whereas quinolones (enrofloxacin and marbofloxacin) and β-lactams (penicillin G, amoxicillin, ampicillin, cefquinome and ceftiofur) showed less frequent values (Figure 4). Low correlation was observed between each pair of antimicrobials with the exception of enrofloxacin and marbofloxacin MIC values (R = 0.99), amoxicillin and ampicillin (R = 0.92) and finally, ceftiofur and cefquinome (R = 0.89). Intermediate correlation was observed for amoxicillin/ampicillin with penicillin G (R = 0.62–0.76) and with ceftiofur/cefquinome (R = 0.69–0.72). Interestingly, a correlation value of 0.69 and 0.47 for penicillin and ceftiofur and penicillin and cefquinome, respectively, was obtained. *S. suis* isolates were grouped into 14 clusters (Figure S4 in Supplementary Materials) according to their MIC values, but most of the isolates (65.3%) were grouped in four clusters and the remaining five clusters included only 5.4% of the strains.

#### 2.3.5. *Escherichia coli*

**Figure 5:**
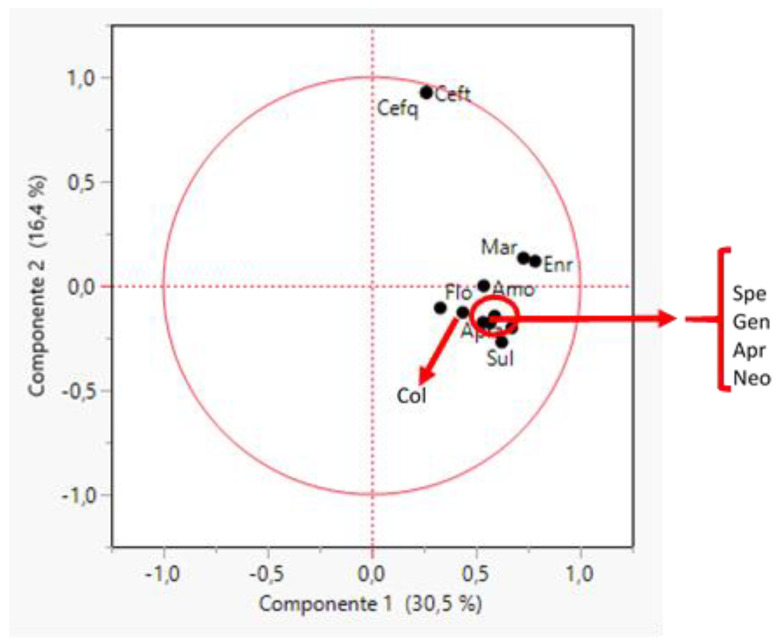
Biplot obtained through a principal component analysis of MIC values for twelve antimicrobials and 1571 strains of *Escherichia coli* (EC). The antimicrobials tested were amoxicillin (Amo), apramycin (Apr), cefquinome (Cefq), ceftiofur (Ceft), colistin (Col), enrofloxacin (Enr), florfenicol (flo), gentamycin (Gen), marbofloxacin (Mar), neomycin (Neo), spectinomycin (Spe) and sulfamethoxazole/trimethoprim (Sul).

The first two principal components could explain 46.9% of the variance for the MIC values against all the tested antimicrobials (Figure 5). All the antimicrobials tested showed very variable MIC values locating all of them far from the origin of the axes in the plot, particularly cephalosporins and quinolones. A low correlation between each pair of antimicrobials was observed, with the exception of enrofloxacin and marbofloxacin MIC values (R = 0.82) and ceftiofur and cefquinome (R = 0.92). *E. coli* isolates were grouped into 18 clusters (Figure S5 in Supplementary) according to their MIC values. Thus, the number of isolates by cluster was mostly between 5% and 11% of the total.

## 3. Discussion

Preventive medicine programs for livestock must be based on controlling diseases with measures such as external and internal biosecurity, use of vaccines, hygiene and disinfection, management measures (e.g., all in–all out procedures) and good husbandry protocols (e.g., optimal temperature and ventilation) to optimize welfare of the animals [22]. These measures will be able to reduce the use of antibiotics and therefore, be one of the drivers for the generation of AMR bacteria not only in animals, but also in humans and the environment following a one-health approach [23]. In any case, the use of antibiotics is necessary to guarantee animal welfare and good veterinary practices once the disease appears in the farm. However, the optimization of antibacterial therapy is crucial in human and veterinary medicine in order to reduce the selection of resistant bacteria [24,25]. Our cluster analysis clearly demonstrates a great variability for the MIC pattern in all the porcine pathogens studied. These results reinforced the recommendation of determining AST for each clinical case in order to select the most suitable drug in a case-by-case situation according to the new European legislation. As discussed in our previous paper [17], an antimicrobial stewardship for a case-by-case situation is possible if an epidemiological link is proposed, meaning that within the same production system (same farm or inter-related farms), the microbiological diagnosis and the determination of its antimicrobial susceptibility could be used to treat similar clinical cases caused by this particular bacterium during a set period of time.

The first step to optimize the use of antibiotics in livestock is to carry out a good diagnosis including AST [19]. The existence of CBPs for each pair antimicrobial/pathogen is critical to correctly foresee the clinical outcome for each clinical case. In this study, we have not carried out an analysis based on the phenotype (resistant/susceptible) according to CBPs, due to the absence of standardized CBPs for many antimicrobials and bacterial species, such as *B. bronchiseptica*, *S. suis* or *E. coli* [6,7,26,27]. However, we have performed a principal component analysis to decipher the MIC pattern for each included pathogen. This multivariate methodology allows analyzing relationships within a set of numerical variables, without any previous assumptions on data distribution. We have been unable to observe any association between the MIC patterns for different antimicrobial families (e.g., quinolones versus macrolides) suggesting that, co-selection mechanisms are not generally present in these porcine pathogens. However, co-selection mechanisms for some pig pathogens have been described in the literature, mainly based on the presence of plasmids harboring antimicrobial resistance genes against several antimicrobial families [28,29,30]. These cases could be also present in our database but our analysis, including all the isolates and antimicrobials, was unable to detect them.

We have observed a high correlation and a similar MIC distribution between the MIC values for marbofloxacin and enrofloxacin, third and second generation of fluoroquinolones, respectively, for all the studied pathogens (*E. coli*, *S. suis*, APP, *P. multocida* and *B. bronchiseptica*) suggesting that a cross-resistance mechanism could be present for fluoroquinolones and all the porcine pathogens studied. Fluoroquinolones are known to have two enzyme targets in the bacterial cell, DNA gyrase and topoisomerase IV, both involved in DNA replication. Mutations in key sites nominated quinolone resistance-determining region (QRDR) of the gyrase or topoisomerase IV can decrease the binding affinity for the antimicrobial [31]. Studies have shown that isolates with a single mutation in the QRDR to some extent are refractory to the bactericidal effect of fluoroquinolones, and in Gram-negative organisms, once a first-step mutation has reduced the susceptibility, further mutations increase the resistance and therefore the MIC value [32]. For instance, in *S. suis*, cross-resistance to second- and third-generation fluoroquinolones have been associated with two modifications in GyrA (at positions S81 and E85 of GyrA) and one in ParC (at position S79) [33]. Plasmid-mediated resistance and efflux pumps have also been described to confer resistance to these drugs [34]. Independently of the exact mechanisms of cross-resistance for porcine pathogens, from our data it is possible to conclude that testing one quinolone (enrofloxacin or marbofloxacin) is enough to carry out AST for this antimicrobial family and these porcine pathogens. This affirmation should be supported by other research groups using other quinolones such as danofloxacin in future studies. 

While it was not possible to test cross-resistance between ceftiofur and cefquinome for APP, *P. multocida* and *B. bronchiseptica,* because only ceftiofur was in the panel, a high correlation and a similar MIC distribution was observed between ceftiofur and cefquinome MIC values in *E. coli* and *S. suis.* These results suggest that the same mechanism of resistance or cross-resistance could be present between third- and fourth-generation cephalosporins for each of these two pathogens. Similar results can be extracted from data published by other researchers for *E. coli* [35,36]. In *E. coli*, resistance to cephalosporins most generally is associated with the presence of genes encoding for extended spectrum beta-lactamases (ESBL) or AmpC enzymes. Some of the most common resistance genes in livestock are variants of *bla*_TEM_, *bla*_CTX-M_, *bla*_SHV_ and *bla*_CMY_ [37,38]. These genes are generally located in plasmids and are known to cause cross-resistance between different cephalosporins [39].

The correlation observed between the MIC value for beta-lactams and *S. suis* must be carefully studied and discussed. In Streptococci, a typical route to acquire beta-lactam resistance involves variants in the penicillin-binding-protein genes (*pbp*) and it is necessary the joint action of many *pbp* variants to explain significant changes in MIC [40,41]. *S. suis* has three key *pbp* genes (*pbp1A*, *pbp2B* and *pbp2X*). Single point mutations in *pbp2X* alone have small effects on the MIC value, whereas additional mutations in the rest of *pbp*, taking place in a set order, may explain the high MIC value observed for some antimicrobials belonging to this family [42]. On the other hand, studies have shown that cefotaxime, a third-generation cephalosporin like ceftiofur, selectively inactivates *pbp2X* but not *pbp2B* [43]. This finding may explain the intermediate association between MIC value for the combination of penicillin and aminopenicillins and cephalosporins observed in our research work and supports that the mechanisms of resistance for beta-lactams could be only shared by a percentage (50–72%) of the isolates [44,45,46]. However, a high correlation and a similar MIC distribution has been observed between the MIC value for amoxicillin and ampicillin, agreeing perfectly with the CLSI recommendation of testing any of both drugs for Gram-positive bacteria [26]. 

The correlation between the MIC values for the tested tetracyclines (doxycycline and oxytetracycline) is very high for *B. bronchiseptica* and intermediate for the rest of bacteria tested (APP, *S. suis* and *P. multocida*). These results suggest that the mechanisms of resistance for tetracyclines could be common for most of the *B. bronchiseptica* strains, but these mechanisms could be only shared by a percentage (close to 50%) of the APP, *S. suis* and *P. multocida* strains. Binding site mutations in rRNA conferring tetracycline resistance are usually found in bacteria with low rRNA gene copy numbers. This could be the case for *B. brochiseptica*, which have three copies of the 16S rRNA. However, a plasmid-mediated mechanism such as *tet*(A) or *tet*(C) has also been described for this pathogen [14] that could provide this cross-resistant phenotype. In general, three mechanisms of resistance to tetracyclines have been well described, efflux pumps, ribosomal protection, and enzymatic inactivation of the drugs [47]. Still further studies should be addressed to identify the specific target and mechanism of action of each one of the drugs included in this family, which nowadays remain poorly understood [48]. 

It is especially interesting the absence of cross-resistance between families with high number of drugs such as aminoglycosides and macrolides. Thus, resistance mechanisms are complex and differ between aminoglycoside molecules and between bacterial species, and generally, there is less cross-resistance when compared with other classes of antimicrobials [49]. In the case of macrolides, the level of cross-resistance depends on the mechanisms conferring the resistance. Thus, it is well known that modification of the ribosomal target probably confers cross-resistance to macrolides and lincosamides as a whole family, whereas efflux pumps and enzymatic inactivation do not and, as a consequence, a lot of variability in cross-resistance is expected [50]. This result implies that it is not possible to select “target” drugs for macrolides and aminoglycosides as surrogate markers for all the family with the only exception of tildipirosin/tulathromycin and *P. multocida.* This result could be extremely relevant to set up European programs for surveillance of antimicrobial resistance of porcine pathogens [20].

## 4. Materials and Methods

### 4.1. Clinical Samples

Between 2018 and 2020, samples were taken from diseased or recently deceased pigs from farms showing acute clinical signs of respiratory tract infections, clinical symptoms compatible with *S. suis* infections (nervous symptoms and arthritis) or pigs showing diarrhea that had not been exposed to antimicrobial treatment for, at least, 15 days prior to sampling. Thus, the sampled animals were between 3 and 24 weeks old for animals showing overt respiratory symptoms with or without depression and/or hyperthermia (>39.8 °C). For each clinical case, samples of lungs of two recently deceased pigs (<12 h) were submitted under refrigeration to the laboratory. On the other hand, the sampled animals were between 3 and 12 weeks old for animals showing clinical symptoms compatible with *S. suis* infections (nervous symptoms and arthritis) and pigs showing diarrhea. In the case of *S. suis* infections, whole blood, cerebrospinal fluid or articular fluid were obtained from sick or recently deceased animals (<12 h). Finally, intestinal content obtained from humanly euthanized animals or watery diarrhea from sick pigs were obtained from animals showing digestive symptoms during the post-weaning period. In both cases, the samples were submitted under refrigeration to the laboratory and processed during the following 24 h after collection.

### 4.2. Bacterial Isolation and Identification

Clinical specimens were cultured aseptically onto blood agar (Columbia agar with 5% Sheep blood, 254005 BD), chocolate agar (GC II agar with IsoVitaleX, 254060, BD or blood Agar No. 2 Base, 257011, BD) and MacConkey agar (4016702, Biolife Italiana Srl) and incubated at 35 ± 2 °C in aerobic conditions with 5–10% CO_2_ for 24–48 h to address the isolation of respiratory and systemic pathogens. Finally, for the isolation of digestive pathogens, specimens were cultured aseptically onto Blood agar, MacConkey agar and Xylose-Lysine-Desoxycholate Agar (XLD, CM0469, Oxoid). The plates were incubated at 35 ± 2 °C in aerobic conditions for 24 h.

Identification of isolates for respiratory pathogens (APP, *P. multocida* and *B. bronchiseptica*), *S. suis* and digestive pathogens was carried out by matrix-assisted laser desorption ionization-time of flight (MALDI-TOF Biotyper System, Bruker Daltonics, Bremen, Germany) as previously described [17]. Individual strains were stored at −80 °C in brain heart infusion (CM1135, Oxoid) with 30% of glycerol (G9012, Sigma-aldrich, St. Louis, MO, USA). 

### 4.3. Antimicrobial Susceptibility Testing

MIC values were determined using the broth microdilution method by means of customized 96-well microtiter plates (Sensititre, Trek diagnostic Systems Inc., East Grinstead, UK) containing a total of twelve and seven–eight antibiotics/concentrations, respectively, in accordance with the recommendations presented by the Clinical and Laboratory Standards Institute [26,27]. This antimicrobial panel was selected to represent commonly used compounds for treatment of pig diseases in practice. 

Bacteria were thawed, cultured on chocolate agar or blood agar, and incubated at 35 ±2 °C in ambient air (or with 5–10% CO_2_ for APP) for 18–24 h. Three to five colonies were picked and emulsified in demineralized water (or Cation Adjusted Muëller–Hinton Broth (CAMHB) for APP and *S. suis*) to obtain a turbidity of 0.5 McFarland standard (Sensititre™ nephelometer V3011). Suspensions were further diluted in CAMHB for *E. coli*, CAMHB or Cation Adjusted Mueller–Hinton Broth with 2.5–5% Lysed Horse Blood for *P. multocida* and *B. bronchiseptica*, Cation Adjusted Mueller Hinton Broth with 2.5–5% Lysed Horse Blood (CAMHB + LHB) for *S. suis* and Veterinary Fastidious Medium (VFM) or Mueller–Hinton Fastidious broth with Yeast (MHF-Y) for APP to reach a final inoculum concentration of 5 × 10^5^ cfu/mL. Then, the Sensititre panel was reconstituted by adding 100 μL/well of the inoculum. Plates containing *E. coli* and *B. bronchiseptica* isolates were incubated at 35 ± 2 °C for 16–20 h, *P. multocida* isolates were incubated at 35 ± 2 °C for 18–24 h and *S. suis* isolates were incubated at 35 ± 2 °C for 20–24 h. In the case of APP isolates, plates were covered with a perforated seal and incubated at 35 ± 2 °C with 5–10% CO_2_ for 20–24 h. 

The antibiotic panels were read manually using Sensititre™ Vizion (V2021) and the MIC value was established as the lowest drug concentration inhibiting visible growth. For each strain tested, a colony count and a purity check were performed following CLSI and manufacturer recommendations. Moreover, quality control strains were also included. Thus, *Actinobacillus pleuropneumoniae* (ATCC 27090™), *Escherichia coli* (ATCC 25922™), *Streptococcus pneumoniae* (ATCC 49619™) and *Enterococcus faecalis* (ATCC 29212™) were included as quality control following CLSI recommendations [26,27]. The MICs of the quality control strains had to be within acceptable CLSI ranges to accept the results obtained in the laboratory. 

### 4.4. Data Analysis

The results of the sensitivity tests are presented as MIC distributions (MIC range, MIC_50_ and MIC_90_) and these were determined for each species–antimicrobial combination. Moreover, a principal component analysis (PCA) was carried out. This multivariate technique has been used to decipher the antimicrobial susceptibility pattern for each bacterium taking into account the MIC values for all the drugs. PCA also provides information about correlations between variables with matrix correlations. The correlation between MIC values for each pair of antimicrobials was classified as high (>0.8), intermediate (0.5–0.8) and low (<0.5) for each bacterium. Finally, a constellation plot was also generated using between-group linkage via Ward’s hierarchical clustering that allows generating clusters of strains of each studied pathogen according to their antimicrobial susceptibility testing for all the antimicrobials together. All the data analysis was carried out with JMP^®^, Version 13 (SAS Institute Inc., Cary, NC, USA, 1989–2019).

## 5. Conclusions

In general, antimicrobial susceptibility testing results from one drug are not representative from the whole antimicrobial family, however, for specific drug–bug combinations, MIC values from one drug may be extrapolated to the whole family. This is the case of fluoroquinolones (marbofloxacin and enrofloxacin) and all porcine pathogens tested herein, for ceftiofur and cefquinome in combination with *E. coli* and *S. suis*, ampicillin and amoxicillin with *S suis*, tetracyclines (doxycycline and oxytetracycline) with *B. bronchiseptica* and tildipirosin/tulathromycin with *P. multocida*.

## Figures and Tables

**Table 1:** *Actinobacillus pleuropneumoniae* (A), *Pasteurella multocida* (B) and *Bordetella bronchiseptica* (C) MIC range, MIC_50_ and MIC_90_ for 490 APP, 285 PM and 73 BB strains, respectively, isolated from respiratory clinical cases. The same color in the antimicrobial column represents member of the same antimicrobial family.

A: *Actinobacillus pleuropneumoniae*			
Antimicrobial	Range (μg/mL)	MIC_50_ (μg/mL)	MIC_90_ (μg/mL)
Amoxicillin	0.06–16	0.5	8
Ceftiofur	0.06–0.25	0.06	0.06
Doxycycline	0.12–16	2	4
Enrofloxacin	0.03–4	0.06	0.5
Florfenicol	0.06–8	0.25	0.5
Marbofloxacin	0.03–4	0.03	0.5
Oxytetracycline	0.12–8	8	8
Sulfamethoxazole/trimethropim ^&^	0.06–32	0.12	2
Tiamulin	2–32	16	16
Tildipirosin	1–64	8	8
Tilmicosin	4–64	16	16
Tulathromycin	8–64	32	64
B: *Pasteurella multocida*			
Amoxicillin	0.12–8	0.25	0.5
Ceftiofur	0.06–1	0.06	0.12
Doxycycline	0.12–16	0.5	2
Enrofloxacin	0.03–1	0.03	0.06
Florfenicol	0.12–8	0.5	0.5
Marbofloxacin	0.03–1	0.03	0.12
Oxytetracycline	0.12–8	1	8
Sulfamethoxazole/trimethropim ^&^	0.03–8	0.12	4
Tiamulin	2–64	16	32
Tildipirosin	0.5–64	2	4
Tilmicosin	1–64	8	16
Tulathromycin	0.5–64	2	4
C: *Bordetella bronchiseptica*			
Amoxicillin	8–16	8	16
Ceftiofur	4	4	4
Doxycycline	0.12–4	1	2
Enrofloxacin	0.12–4	0.5	1
Florfenicol	1–8	4	4
Marbofloxacin	0.12–2	0.5	1
Oxytetracycline	0.25–8	2	4
Sulfamethoxazole/trimethropim ^&^	0.06–8	4	4
Tiamulin	32–64	64	64
Tildipirosin	0.5–16	8	8
Tilmicosin	8–64	32	64
Tulathromycin	2–16	8	16

^&^ The MIC value is showed for trimethoprim in the combination sulfamethoxazole/trimethropim.

**Table 2:** *Streptococcus suis* MIC range, MIC_50_ and MIC_90_ for 398 strains isolated from systemic clinical cases. The same color in the antimicrobial column represents member of the same antimicrobial family.

Antimicrobial	Range (μg/mL)	MIC_50_ (μg/mL)	MIC_90_ (μg/mL)
Amoxicillin	0.06–8	0.06	0.5
Ampicillin	0.06–4	0.06	0.5
Ceftiofur	0.06–4	0.25	1
Cefquinome	0.06–4	0.06	0.25
Doxycycline	0.12–16	8	16
Enrofloxacin	0.03–4	0.5	1
Florfenicol	1–8	2	4
Marbofloxacin	0.03–4	1	2
Penicillin G	0.06–8	0.25	2
Sulfamethoxazole/trimethropim ^&^	0.06–4	1	4
Tiamulin	0.5–64	2	32
Tilmicosin	4–64	64	64

^&^ The MIC value is showed for trimethoprim in the combination sulfamethoxazole/trimethropim.

**Table 3:** *Escherichia coli* MIC range, MIC_50_ and MIC_90_ for 1571 strains isolated from digestive clinical cases. The same color in the antimicrobial column represents member of the same antimicrobial family.

Antimicrobial	Range (μg/mL)	MIC_50_ (μg/mL)	MIC_90_ (μg/mL)
Amoxicillin	0.12–16	8	16
Apramycin	0.5–64	4	32
Ceftiofur	0.06–8	0.5	8
Cefquinome	0.06–8	0.12	8
Colistin	0.25–32	1	4
Enrofloxacin	0.03–4	1	4
Florfenicol	1–8	8	8
Gentamycin	0.12–64	1	16
Marbofloxacin	0.03–4	0.5	4
Neomycin	0.5–64	4	64
Spectinomycin	4–128	128	128
Sulfamethoxazole/trimethropim ^&^	0.03–8	4	8

^&^ The MIC value is showed for trimethoprim in the combination sulfamethoxazole/trimethropim.

## Data Availability

The data presented in this study are available on reasonable request from the corresponding author. The data are not publicly available due to confidentiality issues related with clinical cases.

## Supplementary Materials

The following supporting information can be downloaded at: https://www.mdpi.com/article/10.3390/antibiotics11050638/s1, Figure S1. Constellation plot of the 490 strains of *Actinobacillus pleuropneumoniae* (APP) after a hierar-chical clustering analysis of MIC values for amoxicillin (Amo), ceftiofur (Ceft), doxycycline (Dox), enrofloxacin (Enr), florfenicol (flo), marbofloxacin (Mar), oxytetracycline (Oxy), sulfamethoxazole/trimethoprim (Sul), tiamulin (Tia), tildipirosin (Tild), tilmicosin (Tilm) and tulathromycin (Tul); Figure S2. Constellation plot of the 285 strains of *Pasteurella multocida* (PM) after a hierarchical clustering analysis of MIC values for amoxicillin (Amo), ceftiofur (Ceft), doxycycline (Dox), enrofloxacin (Enr), florfenicol (flo), marbofloxacin (Mar), oxytetracycline (Oxy), sulfamethoxazole/trimethoprim (Sul), tiamulin (Tia), tildipirosin (Tild), tilmicosin (Tilm) and tulathromycin (Tul); Figure S3. Constellation plot of the 78 strains of *Bordetella bronchiseptica* (BB) after a hierarchical clustering analysis of MIC values for amoxicillin (Amo), ceftiofur (Ceft), doxycycline (Dox), enrofloxacin (Enr), florfenicol (flo), marbofloxacin (Mar), oxytetracycline (Oxy), sulfamethoxazole/trimethoprim (Sul), tiamulin (Tia), tildipirosin (Tild), tilmicosin (Tilm) and tu-lathromycin (Tul); Figure S4. Constellation plot of the 398 strains of *Streptococcus suis* after a hierarchical clustering analysis of MIC values for ampicillin (Amp), amoxicillin (Amo), cefquinome (Cef), ceftiofur (Ceft), doxycycline (Dox), enrofloxacin (Enr), florfenicol (flo), marbofloxacin (Mar), Penicillin G, sulfamethoxazole/trimethoprim (Sul), tiamulin (Tia), and, tilmicosin (Tilm); Figure S5. Constellation plot of the 1571 strains of *Escherichia coli* (EC) after a hierarchical clustering analysis of MIC values for amoxicillin (Amo), apramycin (Apr), cefquinome (Cefq), ceftiofur (Ceft), colistin (Col), enrofloxacin (Enr), florfenicol (flo), gentamycin (Gen), marbofloxacin (Mar), neomycin (Neo), spectinomycin (Spe) and sulfamethoxazole/trimethoprim (Sul).
