# Supplementary material for: Antimicrobial Susceptibility Testing of Porcine Bacterial Pathogens: Investigating the Prospect of Testing a Representative Drug for Each Antimicrobial Family
Source: Antibiotics (Basel). 2022 May 10;11(5):638. doi: 10.3390/antibiotics11050638 (PMC9137606; doi:10.3390/antibiotics11050638)
Supplement: Supplementary file 1 [file antibiotics-11-00638-s001.zip › antibiotics-1698461-supplementary.pdf]

antibiotics

MDPI

Supplementary Materials

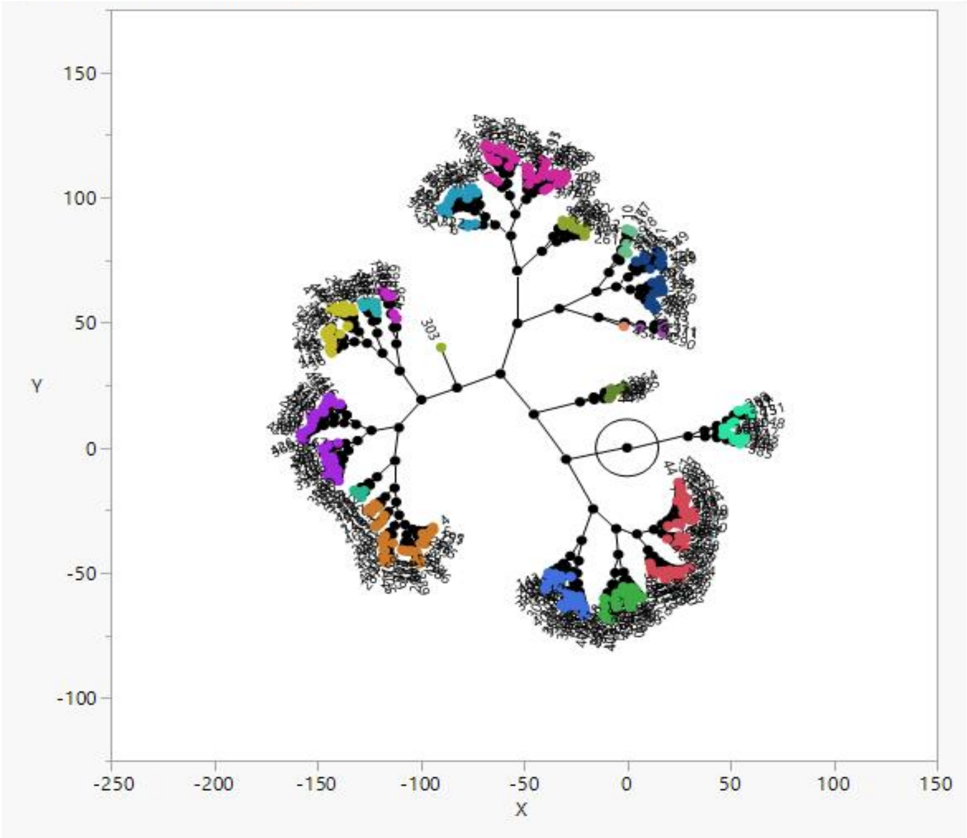

Figure S1. Constellation plot of the 490 strains of *Actinobacillus pleuropneumoniae* (APP) after a hierarchical clustering analysis of MIC values for amoxicillin (Amo), ceftiofur (Ceft), doxycycline (Dox), enrofloxacin (Enr), florfenicol (flo), marbofloxacin (Mar), oxytetracycline (Oxy), sulfamethoxazole/trimethoprim (Sul), tiamulin (Tia), tildipirosin (Tild), tilmicosin (Tilm) and tulathromycin (Tul).

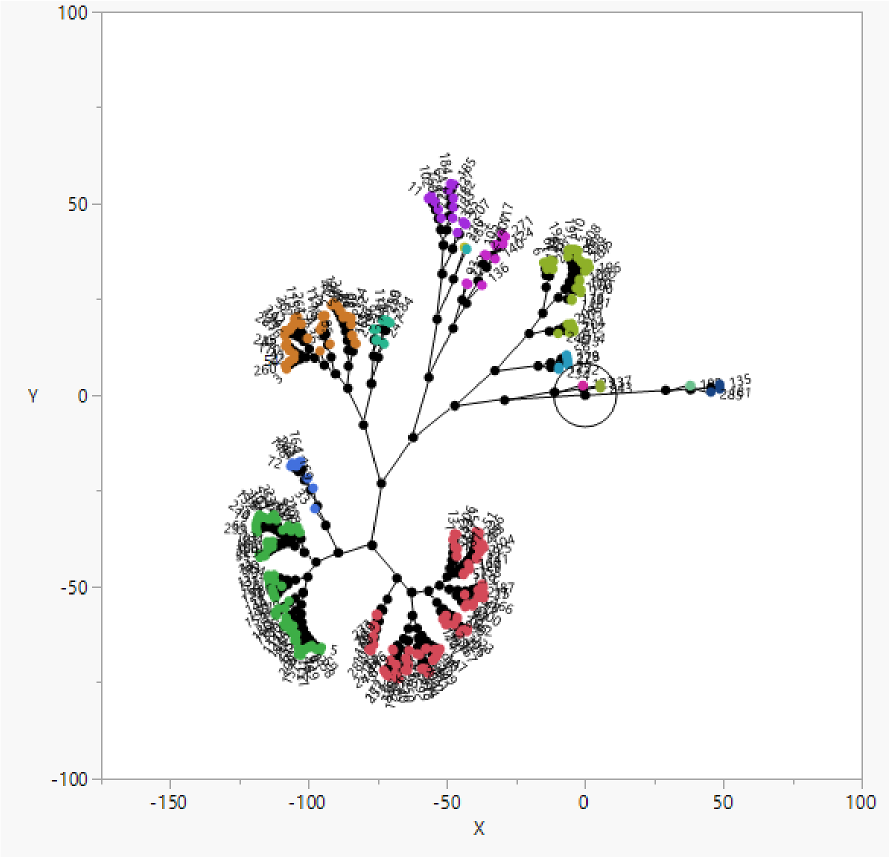

Figure S2. Constellation plot of the 285 strains of *Pasteurella multocida* (PM) after a hierarchical clustering analysis of MIC values for amoxicillin (Amo), ceftiofur (Ceft), doxycycline (Dox), enrofloxacin (Enr), florfenicol (flo), marbofloxacin (Mar), oxytetracycline (Oxy), sulfamethoxazole/trimethoprim (Sul), tiamulin (Tia), tildipirosin (Tild), tilmicosin (Tilm) and tulathromycin (Tul).

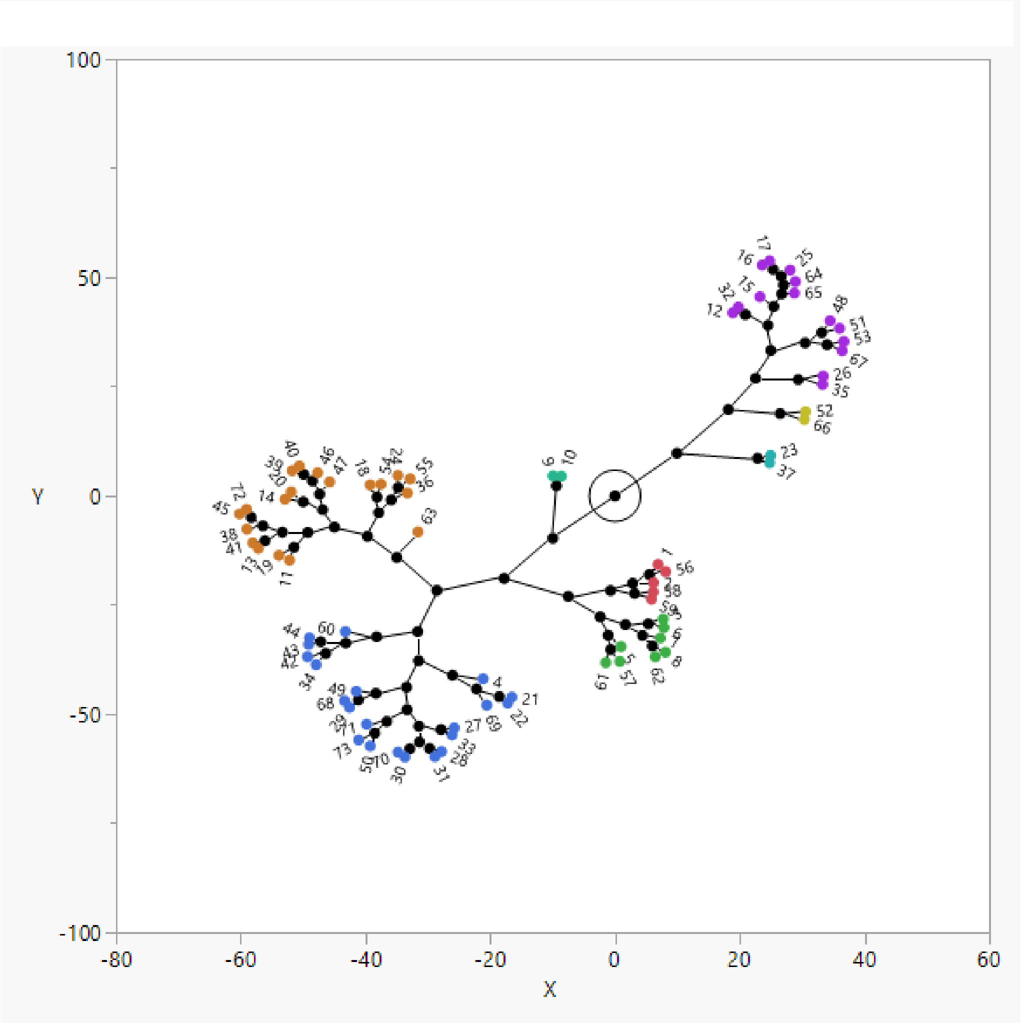

Figure S3. Constellation plot of the 78 strains of *Bordetella bronchiseptica* (BB) after a hierarchical clustering analysis of MIC values for amoxicillin (Amo), ceftiofur (Ceft), doxycycline (Dox), enrofloxacin (Enr), florfenicol (flo), marbofloxacin (Mar), oxytetracycline (Oxy), sulfamethoxazole/trimethoprim (Sul), tiamulin (Tia), tildipirosin (Tild), tilmicosin (Tilm) and tulathromycin (Tul).

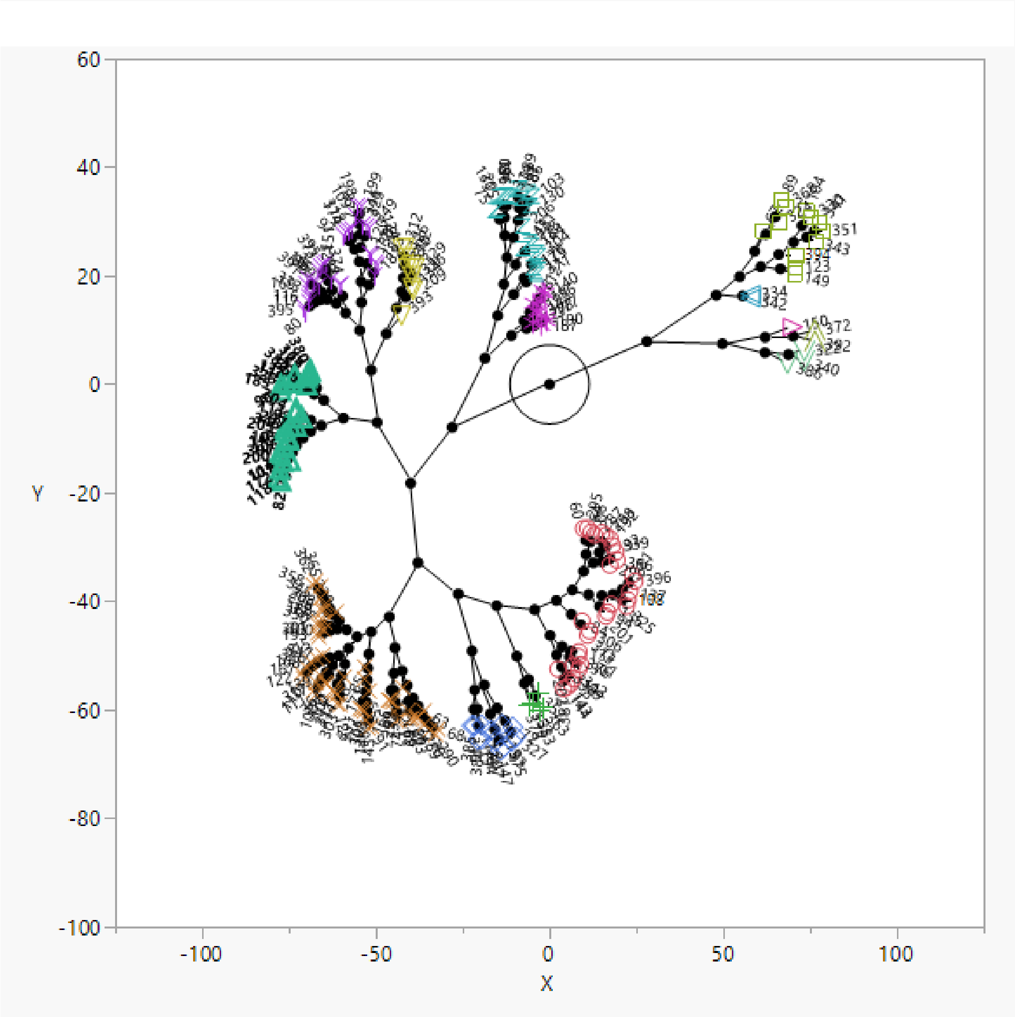

Figure S4. Constellation plot of the 398 strains of *Streptococcus suis* after a hierarchical clustering analysis of MIC values for ampicillin (Amp), amoxicillin (Amo), cefquinome (Cef), ceftiofur (Ceft), doxycycline (Dox), enrofloxacin (Enr), florfenicol (flo), marbofloxacin (Mar), Penicillin G, sulfamethoxazole/trimethoprim (Sul), tiamulin (Tia), and, tilmicosin (Tilm).

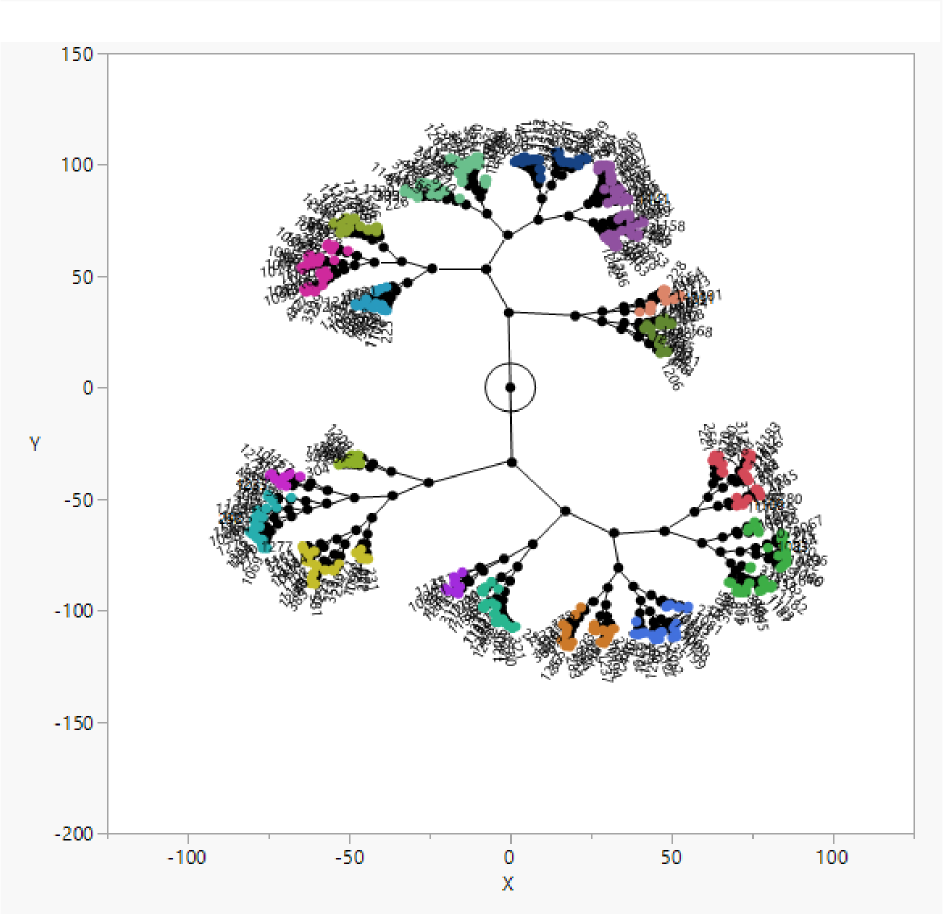

Figure S5. Constellation plot of the 1571 strains of *Escherichia coli* (EC) after a hierarchical clustering analysis of MIC values for amoxicillin (Amo), apramycin (Apr), cefquinome (Cefq), ceftiofur (Ceft), colistin (Col), enrofloxacin (Enr), florfenicol (flo), gentamycin (Gen), marbofloxacin (Mar), neomycin (Neo), spectinomycin (Spe) and sulfamethoxazole/trimethoprim (Sul).
